# Exosomes from Adipose-Derived Stem Cells Alleviate Dexamethasone-Induced Bone Loss by Regulating the Nrf2/HO-1 Axis

**DOI:** 10.1155/2023/3602962

**Published:** 2023-02-01

**Authors:** Xue-wei Yao, Zhi-yi Liu, Neng-feng Ma, Wen-kai Jiang, Zhi Zhou, Bing Chen, Wen-gang Guan, Jun-jie Yan, Min Yang

**Affiliations:** Department of Trauma Orthopedics, The First Affiliated Hospital of Wannan Medical College, Yijishan Hospital, No. 2, Zheshan Xi Road, Anhui 241001 Wuhu, China

## Abstract

The widespread use of therapeutic glucocorticoids has increased the incidences of glucocorticoid-induced osteoporosis (GIOP). Oxidative stress and mitochondrial dysfunction are major causes of GIOP; therefore, alleviation of excess oxidative stress in osteoblasts is a potential therapeutic strategy for osteoporosis. Exosomes derived from ADSCs (ADSCs-Exos), as novel cell-free therapeutics, can modulate various biological processes, such as immunomodulation, reduce oxidative damage, and promote tissue repair as well as regeneration. In this study, ADSCs-Exos restored the viability and osteogenic potential of MC3T3-E1 cells by attenuating apoptosis, oxidative damage, intracellular ROS generation, and mitochondrial dysfunction. Moreover, after pretreatment with ADSCs-Exos, Nrf2 expressions were upregulated in Dex-stimulated osteoblasts. Inhibitory assays showed that silencing Nrf2 partially eliminated the protective effects of ADSCs-Exos. The rat model assays confirmed that ADSCs-Exos alleviated the Dex-induced increase in oxidation levels, restored bone mass of the distal femur, and increased the expressions of Nrf2 and osteogenic markers in bone tissues. Thus, ADSCs-Exos alleviated apoptosis and oxidative stress by regulating Nrf2/HO-1 expressions after Dex and prevented the development of GIOP in vivo.

## 1. Introduction

Dexamethasone (Dex), a synthetic glucocorticoid, is widely used for treatment of inflammatory and autoimmune diseases [1, 2]. However, long-term high-dose glucocorticoids can reduce bone mass and destroy bone microarchitecture, leading to glucocorticoid-induced osteoporosis (GIOP) and osteonecrosis [3]. About 40% of patients on long-term glucocorticoid therapy may have osteoporosis [4]. Endogenous glucocorticoid activities can maintain bone homeostasis [5]. In contrast, exogenous glucocorticoids may induce oxidative stress by promoting the generation of oxidative DNA, destruction of the antioxidative stress system, and excess accumulation of reactive oxygen species (ROS), which are the main mechanisms in GIOP pathogenesis [6]. Excess ROS can reduce the activities of antioxidant enzymes and promote lipid peroxidation, which induces osteoblast apoptosis and inhibits bone formation [7, 8]. Nuclear factor erythroid 2-related factor 2 (Nrf2), which is immediately downstream of ROS, promotes the expressions of antioxidant response element- (ARE-) dependent genes to maintain cellular redox homeostasis, protect cells from apoptosis as well as oxidative stress damage, and balance oxidative mediators, thereby alleviating the development of Dex-induced osteoporosis [9–11]. Therefore, elucidation of the preventive mechanisms of oxidative stress-induced apoptosis will inform the development of evidence-based treatment strategies for long-term Dex-induced osteoporosis.

As mesenchymal cells, adipose-derived stem cells (ADSCs) are widely used as a source of cells for bone regeneration because of their rich functionality and accessibility [12, 13]. However, their low survival rates after transplantation is a major challenge in clinical practice [14]. Mesenchymal stem cells participate in intercellular signaling by secreting paracrine factors, rather than direct regulation [15, 16]. Exosomes have a crucial effect in the paracrine mechanism [17, 18].

Exosomes (30-150 nm in diameter), which are also known as extracellular vesicles, are secretory vesicles formed by the endocytosis-fusion-efflux process of cells. They contain abundant cytoplasmic proteins, lipids, and genetic factors for intercellular communication and cell differentiation [19, 20]. Exosomes derived from ADSCs (ADSCs-Exos) exhibit therapeutic effects like those of their parent stem cells because they contain similar bioactive substances [21]. Shen et al. reported that ADSCs-Exos can attenuate lipopolysaccharide- (LPS-) induced inflammatory responses and ameliorate sepsis as well as multiorgan injury by activating Nrf2/HO-1 [22]. Moreover, ADSCs-Exos promote endothelial cell proliferation as well as angiogenesis and accelerate wound healing in diabetic foot ulcers patients, especially when Nrf2 is overexpressed [23]. However, it has not been established whether ADSCs-Exos can activate Nrf2/HO-1 to alleviate dexamethasone-induced apoptosis and dysfunction in osteoblasts.

There is a need to conclusively define the efficacy and potential mechanisms of ADSCs-Exos on oxidative damage of osteoblasts. In this study, we characterized ADSCs-Exos and studied their effects on viability, apoptosis, oxidative stress, ROS aggregation, and mitochondrial membrane potential of MC3T3-E1 cells. Moreover, the possible mechanisms via which ADSCs-Exos function were investigated. Finally, the therapeutic roles of ADSCs-Exos in GIOP rat models were clarified. Our findings provide a new theoretical basis and show that ADSCs-Exos are promising cell-free therapeutic agents for GIOP.

## 2. Materials and Methods

### 2.1. Isolation and Identification of ADSCs

ADSCs were isolated and cultured based on previous experience [24]. Three- to four-week-old SD rats were sterilized in 75% alcohol for 30 min after cervical dislocation execution. Adipose tissues in bilateral inguinal fat pads were collected, rinsed with sterile phosphate-buffered saline (PBS), and sliced into 1 mm^3^ pieces. The tissue was digested using type I collagenase for 60 min (Solarbio, China) with vibrations every 10 min. Then, the tissue was centrifuged at 37°C (1200 rpm, 10 min). Precipitates were resuspended in a high-sugar medium supplemented with 10% FBS and 1% penicillin-streptomycin and incubated in 5% CO_2_ at 37°C. The cultural medium was replaced every other day. Then, the cells were identified by detecting the expressions of CD29, CD90, CD34, and CD90 (BioLegend, USA) in 3 passages by flow cytometry (FC500MPL, Beckman Coulter, USA).

### 2.2. Isolation and Characterization of Exosomes

The cells were cultured and maintained to reach 90% confluence in 3 passages after which the original medium was changed to serum-free high-glucose medium. The conditioned medium was collected after 48 h. Exosomes secreted by ADSCs were purified from the conditioned medium by differential centrifugation [25]. Briefly, the conditioned medium was centrifuged at 2000 g for 10 min and 10,000 g for 20 min to remove cell debris. Then, the supernatant was centrifuged at 120000 g for 70 min. The whole process was performed at 4°C. The final precipitate was resuspended in 500 *μ*l PBS to obtain an ADSC-Exo suspension. Total exosome protein concentrations were determined using the BCA Protein Assay Kit (Beyotime, China). We observed their morphologies by nanoparticle tracking analysis (NTA) and transmission electron microscopy (TEM) to measure the concentrations of exosomes and particle size distributions. Then, marker proteins of ADSCs-Exos (CD63, CD81, and HSP-70) were measured.

### 2.3. Exosome Labeling and Uptake

Purified ADSCs-Exos were labeled with PKH26 (MaoKang Biotechnology, China), as instructed by the manufacturer. Briefly, ADSCs-Exos were dissolved in 200 *μ*l PBS and added to the same volume of Diluent C supplemented with 4 *μ*l PKH26 dye. Exosomes were placed in an incubator for 30 min and centrifuged at 120,000 × g for 70 min at 4°C, after which the precipitate was retained. The PKH26-labeled ADSC-Exo solution was resuspended in the medium and cocultured with MC3T3-E1. After one day, the cells were fixed in 4% paraformaldehyde for 30 min and broken in 0.5% Triton X-100 solution for 15 min. Phalloidin was used for cytoskeleton staining while cell nuclei were stained using DAPI for 10 min. Fluorescence was observed by confocal microscopy.

### 2.4. Cell Culture

MC3T3-E1 cells were obtained from Guangzhou Cellcook Biotech Co., Ltd. (Guangzhou, China) and treated as follows: (i) PBS group: the cells were cultured in complete DMEM supplemented with the same volume of PBS; (ii) ADSC-Exo group: the cells were cocultured with 50 *μ*g/ml ADSCs-Exos for one day; (iii) Dex group: the cells were precultured in complete DMEM for one day and then with 50 *μ*M Dex for one day; and (iv) Dex+ADSC-Exo group: the cells were cocultured with 25 or 50 *μ*g/ml ADSCs-Exos for one day, then with 50 *μ*M Dex for one day.

### 2.5. Assessment of Cell Viability

In this assay, 3000 logarithmically grown MC3T3-E1 cells were inoculated in each well of 96 well plates to detect different groups of cell viability using the Cell Counting Kit-8 (CCK-8, Solarbio, USA). At a cell density of 80%, different concentrations of Dex (0, 1, 10, 50, and 100 *μ*mol/l) and ADSCs-Exos were used to treat cells, respectively. In the dark, the cells were coincubated with 10 *μ*l CCK-8 reagent and the OD value of cells determined at 450 nm using a microplate reader (BioTek Instruments, Inc., USA).

### 2.6. Small Interfering RNA (siRNA) Transfection

To silence Nrf2, 1 × 10^6^ cells were transfected with Nrf2 siRNA (RiboBio, China) as instructed by the manufacture. After 48 h of transfection, the cells were incubated with ADSCs-Exos and Dex. Western blot was used to assess the transfection efficiency.

### 2.7. Cell Apoptosis Assay

The MC3T3-E1 cells were inoculated in 6-well plates at a density of 1.0 × 10^5^ cells/well. When they reached 80% confluence, they were incubated with Dex with or without ADSCs-Exos, digested and collected. The Annexin V-FITC/PI Apoptosis Detection Kit (KeyGEN, Nanjing, China) was used to measure the effects of ADSCs-Exos and Dex on apoptosis of MC3T3-E1 cells. Briefly, after cells had been treated in different ways, they were collected with 0.25% trypsin. Then, 100 *μ*l of 1x binding buffer was used to resuspend the cell precipitate, after which 5 *μ*l of Annexin V-FITC and 5 *μ*l of propidium iodide (PI) were added and incubated for 15 min, respectively. The cell suspension was added to 400 *μ*l of 1x binding buffer, transferred to a sterile flow cytometry tube for flow cytometry within 1 h.

### 2.8. Mitochondrial Membrane Potential (MMP) Assay

MC3T3-E1 cells were inoculated in 6-well plates at a density of 1.0 × 10^5^ cells per well. After incubation to 60% confluence, they were incubated with Dex with or without ADSCs-Exos. The MMP detection kit (Beyotime, China) was used to measure MMP levels in different groups of cells following the manufacturer's instructions. Briefly, the cells were coincubated with the JC-1 staining working solution at 37°C for 30 min and rinsed with a precooled JC-1 staining buffer. The cells were visualized by fluorescence microscopy.

### 2.9. ROS Assay

After the MC3T3-E1 cells had been treated with Dex with or without ADSCs-Exos, they were washed using PBS. Intracellular ROS levels were determined using the ROS Assay Kit (KeyGEN, Nanjing, China), as instructed by the manufacturer. Briefly, the cells were treated with 10 *μ*M dichlorodihydro-fluorescein diacetate (DCFH-DA), incubated for 30 min in the dark, and assessed by fluorescence microscopy. Flow cytometry was used to measure the mean fluorescence intensity in cells.

### 2.10. Analysis of Malondialdehyde (MDA) and Superoxide Dismutase (SOD) Levels

MC3T3-E1 cells were inoculated with Dex with or without ADSCs-Exos and lysed on ice for 30 min. MDA and SOD levels were determined using commercial assay kits (Jiancheng, Nanjing, China), following the manufacturers' instructions.

### 2.11. Alkaline Phosphatase (ALP) and Alizarin Red S (ARS) Staining

MC3T3-E1 cells were inoculated in 24-well plates at a density of 5.0 × 10^4^ cells per well. After incubation to 80% confluence, the cells were incubated with Dex with or without ADSCs-Exos. The osteogenic differentiation function of different samples was detected using a BCIP/NBT chromogenic kit (Beyotime, China) on the 7^th^ day and ARS staining kit on the 21^st^ day (Solarbio, China). Then, they were observed under an inverted optical microscope.

### 2.12. Western Blot Assay

After MC3T3-E1 cells had been incubated with Dex with or without ADSCs-Exos, the RIPA lysis buffer (Beyotime, China) was added to each sample and lysed on ice. Alternatively, the Nuclear and Cytoplasmic Protein Extraction Kit (Beyotime, China) was used to separate the nuclear and cytoplasmic protein fractions as instructed by the manufacturer. The mixture was centrifuged at 12,000 rpm for 5 min to obtain the supernatant. The proteins were mixed with sample loading buffer in a 100°C water bath for 5 min. Proteins of different molecular weights were separated using SDS-PAGE and transferred to PVDF membranes. The membranes were blocked and incubated overnight at 4°C with the following primary antibodies: Bax (1 : 8000, Proteintech, USA), Bcl2 (1 : 2000, Affinity, USA), caspase-3 (1: 2000, Affinity, USA), cleaved caspase-3 (1 : 2000, Affinity, USA), RUNX2 (1 : 2000, Affinity, USA), BMP2 (1 : 2000, Affinity, USA), Opn (1 : 2000, Affinity, USA), Nrf2 (1 : 2000, Affinity, USA), HO-1 (1 : 2000, Affinity, USA), Lamin B (1 : 2000, Affinity, USA), and *β*-actin (1 : 10000, Affinity, USA).) Then, they were incubated with a secondary antibody (1 : 8000, A21020, Abbkine, USA) for 2 h, assayed by a Super Signal Detector (Thermo Fisher Scientific, USA) and quantitatively analyzed using the ImageJ software.

### 2.13. Immunofluorescence Assay

The MC3T3-E1 cells from different samples were rinsed using PBS, fixed in 4% paraformaldehyde for 30 min, broken in 0.5% TritonX-100 for 15 min, rinsed using PBS, blocked with 10% goat serum for 60 min at room temperature, incubated with primary antibodies at 4°C for 12 h, incubated with fluorescence conjugated secondary antibodies for 2 h, and imaged by confocal microscopy.

### 2.14. *In Vivo* Assays

Fifty 8-week-old SD rats (200 ± 25 g) were obtained from the Hangzhou Qinglong Mountain Experimental Animal Center and maintained in the Animal Experiment Center of Wannan Medical College (temperature, 21-24°C, 12-h dark/light cycle). There were 4 rats per cage, with a daily adequate supply of food and water. The Laboratory Animal Committee of Wannan Medical College approved this study, and all experiments were performed in accordance with the Guide for the Care and Use of Laboratory Animals.

After 1 week of acclimatization, 35 rats were intraperitoneally administered with 2.5 mg/kg Dex once a day, while 15 rats were injected with the same dose of sterile PBS every day. After 4 weeks, 5 rats were selected from each group to assess osteoporosis by femoral imaging and histology. Thirty SD rats were divided into three groups: Dex group and ADSC-Exo (50 and 100 *μ*g) groups. Another 10 rats were used as the Sham group. The Sham and Dex groups were administered with 100 *μ*l of sterile PBS by tail vein injection, while the ADSC-Exo (50 and 100 *μ*g) groups were given 100 *μ*l of PBS containing 50 *μ*g and 100 *μ*g of ADSCs-Exos, respectively. Then, all rats were executed after 5 weeks to obtain the bilateral femurs.

### 2.15. Analysis of the Microstructure of the Distal Femur

To assess bone microstructure changes in the distal femur, microcomputed tomography (micro-CT) was used. The volume of interest (VOI) was 2 mm near the femoral epiphysis line, and the three-dimensional reconstructed images were generated. Then, bone morphometric parameters, such as bone mineral density (BMD), trabecular separation (Tb.Sp), trabecular number (Tb.N), trabecular thickness (Tb.Th), and bone volume to tissue volume (BV/TV) were determined.

### 2.16. Histology, Immunohistochemistry (IHC), and Immunofluorescence Analysis

The bone tissue was fixed in 10% paraformaldehyde for 48 h, immersed in 10% EDTA decalcifying solution, longitudinally sliced into 4 *μ*m thick sections, and hematoxylin-eosin (HE) and Masson stained. IHC was measured using primary antibodies against Nrf2 and Bmp2. For immunofluorescence staining, slices were incubated with primary antibodies at 4°C for 12 h and thereafter with fluorescence conjugated secondary antibodies for 2 h. Then, the slides were observed by fluorescence microscopy.

### 2.17. TUNEL Staining Analysis

The TUNEL Apoptosis Detection Kit (Beyotime, China) was used to determine apoptosis in bone tissues, as instructed by the manufacturer. Briefly, bone tissue sections were rinsed thrice using PBS, incubated with proteinase K for 30 min, and rinsed thrice using PBS. Bone tissue sections were incubated with 50 *μ*l of TUNEL assay solution for 1 h and observed by fluorescence microscopy.

### 2.18. Statistical Analysis

Data are presented as mean ± standard deviation (mean ± SD) and were analyzed using the GraphPad Prism 7 software (La Jolla, CA, USA). Comparisons of means among groups was performed by one-way ANOVA, followed by the Tukey's test for between group comparisons. *P* ≤ 0.05 was considered statistically significant.

## 3. Results

### 3.1. Dexamethasone Inhibited the Survival and Promoted the Apoptosis of MC3T3-E1 Cells

**Figure 1:**
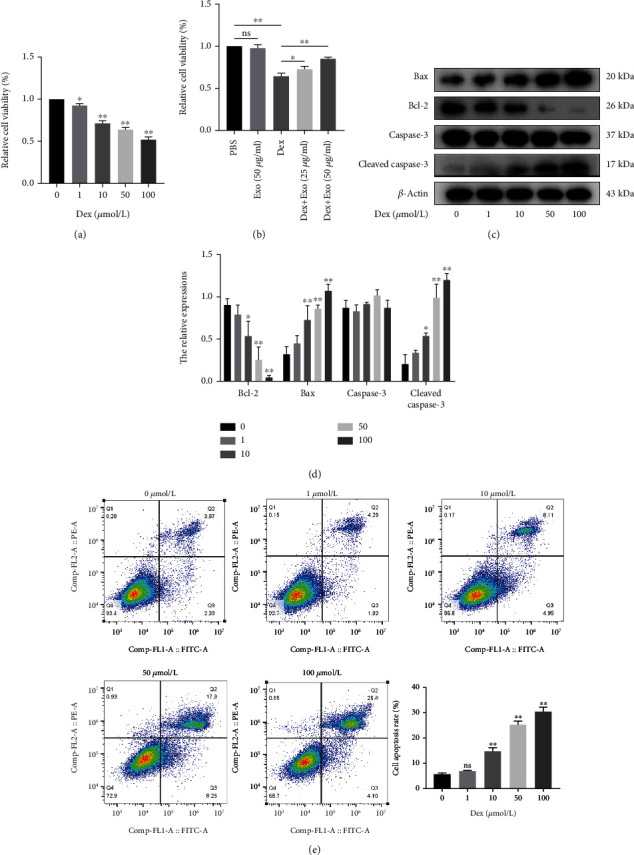
Dexamethasone inhibited survival and promoted apoptosis of MC3T3-E1 cells in a dose-dependent manner. (a) Dex reduced the viability of MC3T3-E1 cells in a dose-dependent manner as detected by CCK-8. (b) ADSCs-Exos alleviated the inhibitory effect of Dex on cell viability. (c, d) Dex downregulated the expression of Bcl-2 and upregulated Bax and cleaved caspase-3 as detected by western blot. (e) Dex dose dependently increased apoptosis as determined by flow cytometry. ^∗^*P* < 0.05 and ^∗∗^*P* < 0.01.

Different concentrations of Dex (0, 1, 10, 50, and 100 *μ*mol/l) dose dependently decreased the viabilities and increased the apoptosis of MC3T3-E1 cells (Figures 1(a) and 1(e)). However, ADSCs-Exos alleviated the inhibitory effect of Dex on cell viability (Figure 1(b)). Dex dose dependently suppressed Bcl-2 levels and elevated the expressions of antiapoptosis proteins (Bax and cleaved caspase-3) (Figures 1(c) and 1(d)). Therefore, 50 *μ*mol/l Dex was used in subsequent experiments.

### 3.2. Characteristics of ADSCs and ADSCs-Exos

**Figure 2:**
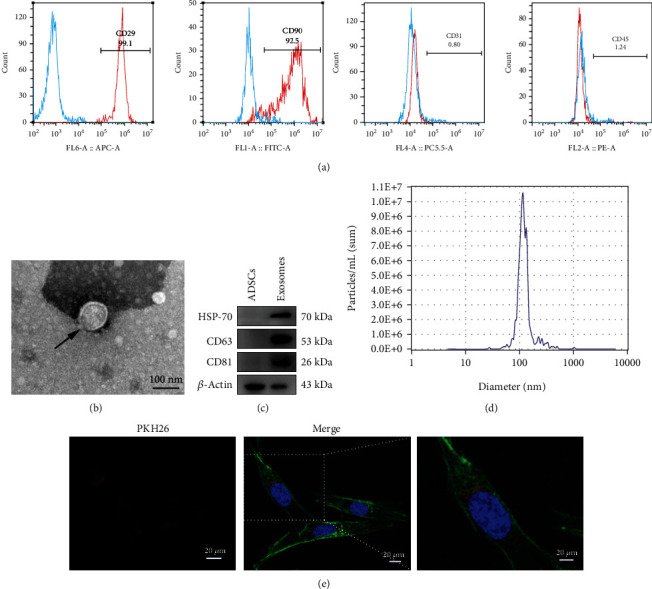
Characteristics of ADSCs and ADSCs-Exos. (a) The results of flow cytometry indicating that the isolated cells were CD31 (0.80%) and CD45 (1.24%) negative, but CD29 (99.10%) and CD90 (92.50%) positive. (b) TEM images of ADSCs-Exos. Scale bar: 100 nm. (c) The western blot results showing that HSP-70, CD63, CD81, and *β*-actin were expressed in ADSCs-Exos. (d) NTA results showing the particle size distribution of ADSCs-Exos (nm). (e) Images showing the uptake of ADSCs-Exos by MC3T3-E1 cells. Blue: nuclei; green: cytoskeleton; red: PKH26-labeled ADSCs-Exos. Scale bar: 20 *μ*m.

The biomarkers of ADSCs were assayed by flow cytometry.  Figure 2(a) shows that the isolated cells were negative for CD31 and CD45 and positive for CD29 and CD90. The TEM assays revealed that ADSCs-Exos were round microvesicles with a diameter of about 100 nm, consistent with NTA result of 115.1 nm (Figures 2(b) and 2(d)). The diameter range of ADSCs-Exos is 80-200 nm in the NTA analysis. Western blot showed that ADSCs-Exos positively expressed the biomarkers of exosomes (HSP70, CD63, and CD81) (Figure 2(c)). Finally, the PHK-26-labeled ADSCs-Exos were cocultured with MC3T3-E1 cells, which showed that ADSCs-Exos could be taken up by MC3T3-E1 cells (Figure 2(e)). These results indicated successful separation of ADSCs-Exos.

### 3.3. ADSCs-Exos Attenuated Dex-Induced Apoptosis in MC3T3-E1 Cells

**Figure 3:**
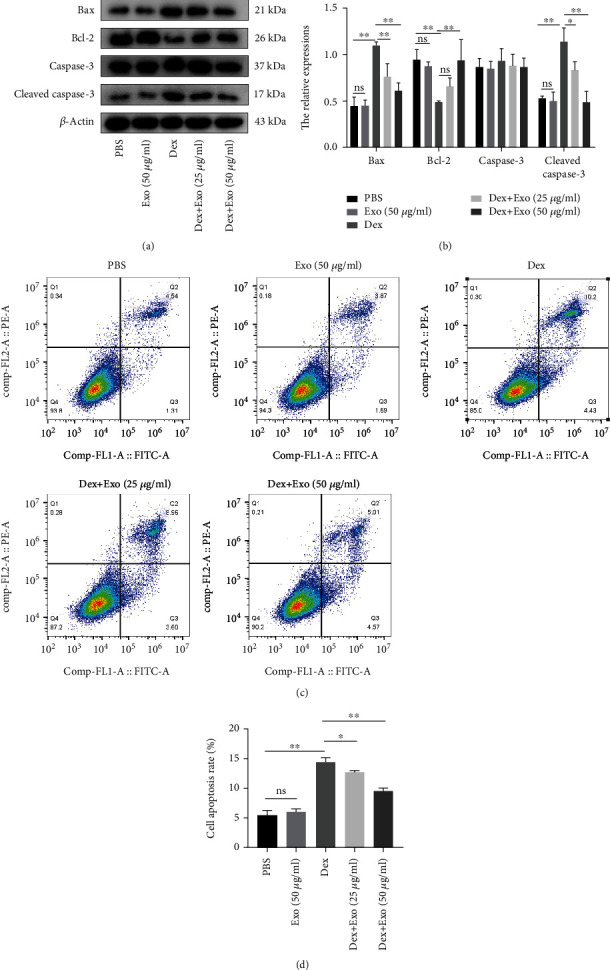
ADSCs-Exos attenuated apoptosis of MC3T3-E1 cells following treatment with Dex. (a) The apoptosis biomarkers in MC3T3-E1 cells in each group were detected by western blot analysis. (b) Quantitative analysis of Bax, Bcl-2, and cleaved caspase-3 expression levels. (c) Flow cytometry showing that ADSCs-Exos inhibited Dex-induced apoptosis. (d) Quantification of apoptosis rate of each group. ^∗^*P* < 0.05 and ^∗∗^*P* < 0.01.

Figures 3(a) and 3(b) show that Dex upregulated proapoptotic Bax and cleaved caspase-3 levels while suppressing antiapoptotic Bcl-2 levels. These proteins were restored to normal levels by ADSC-Exo pretreatment. Consistently, flow cytometry showed that ADSCs-Exos reduced the number of Dex-induced apoptotic cells (Figures 3(c) and 3(d)). In summary, ADSCs-Exos attenuated the Dex-induced cell damage and exerted protective effects on cells.

### 3.4. ADSCs-Exos Suppressed Oxidative Stress after Dex in MC3T3-E1 Cells

**Figure 4:**
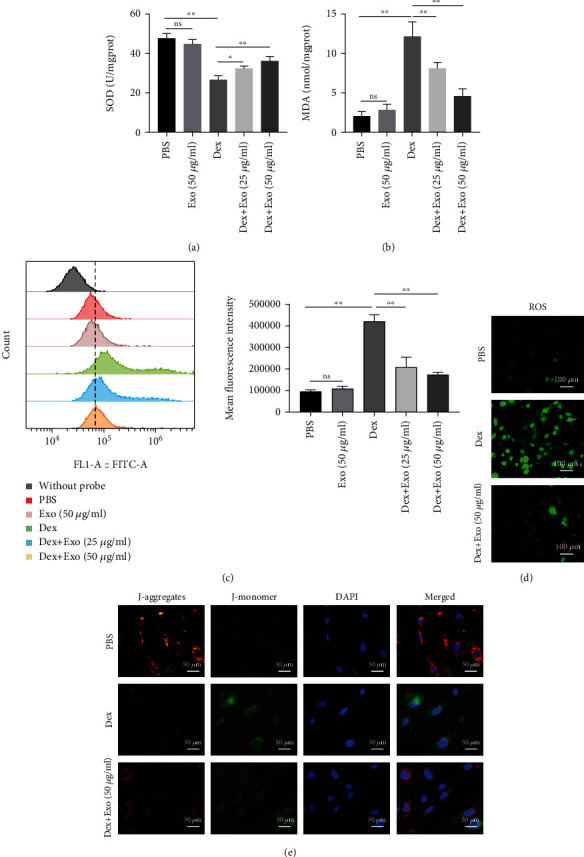
ADSCs-Exos suppressed Dex-induced oxidative stress in MC3T3-E1 cells. (a, b) The activity of SOD and MDA in MC3T3-E1 cells after different treatments. (c) Flow cytometry analysis showing that ADSCs-Exos reversed Dex-induced intracellular ROS accumulation. (d) Representative images showing the fluorescence intensity of ROS in MC3T3-E1 cells after different treatments. (e) Representative images showing JC-1 in PBS, Dex, and Dex+ADSC-Exo groups. ^∗^*P* < 0.05 and ^∗∗^*P* < 0.01.

The molecular mechanisms involved in glucocorticoid-induced osteoporosis include elevated oxidative stress levels and mitochondrial dysfunction in osteoblasts, which results in decreased osteogenic abilities. Therefore, we assessed the effects of ADSCs-Exos on ROS generation and mitochondrial membrane potential in Dex-treated MC3T3-E1 cells. Figures 4(a) and 4(b) show that ADSCs-Exos restored SOD levels and downregulated the activities of MDA, compared to Dex group. Meanwhile, Dex significantly promoted ROS accumulation, and these effects were mitigated by ADSCs-Exos (Figures 4(c) and 4(d)). The decrease in mitochondrial membrane potential is a landmark event for early apoptosis. In physiological states, membrane potential levels in cellular mitochondria are high, and JC-1 aggregates in the mitochondrial matrix to form polymers that produce red fluorescence (J-aggregates). When the mitochondrial membrane potential is low, JC-1 accumulates as a monomer in the mitochondrial matrix and produces green fluorescence (J-monomer). Thus, the mitochondrial membrane potential decreased and green fluorescence was enhanced in the Dex group, whereas after pretreatment with ADSCs-Exos, the mitochondrial membrane potential was restored to near physiological levels (Figure 4(e)).

### 3.5. ADSCs-Exos Restored the Osteogenic Functions of MC3T3-E1 Cells via the Nrf2/HO-1 Axis

**Figure 5:**
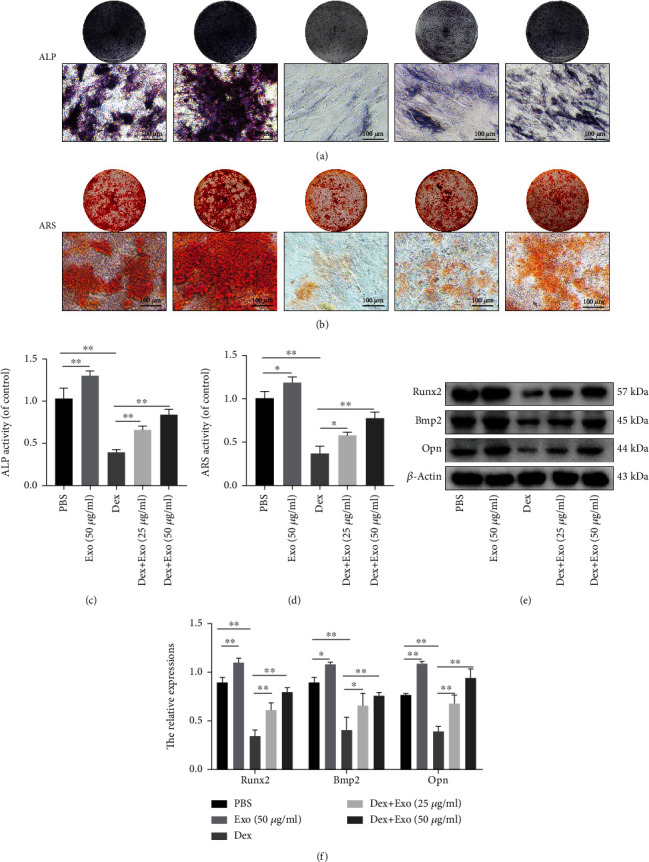
ADSCs-Exos restored the osteogenic function of MC3T3-E1 cells after treatment with Dex. (a, b) Representative images of ALP staining and ARS staining on day 7 and day 21 in MC3T3-E1 cells subjected to different treatments. (c, d) Quantitative analysis of ALP- and ARS-positive area normalized to the control group. (e, f) Expression levels of Runx2, Bmp2, and Opn in MC3T3-E1 cells subjected to different treatments. Scale bar: 100 *μ*m. ^∗^*P* < 0.05 and ^∗∗^*P* < 0.01.

**Figure 6:**
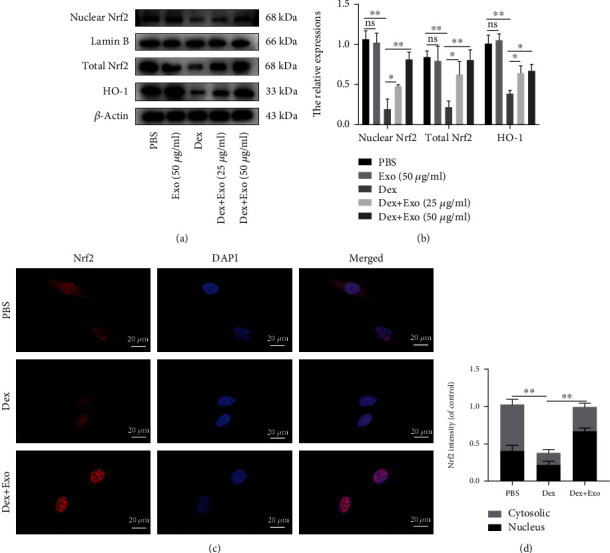
ADSCs-Exos regulated the function of MC3T3-E1 cells via the Nrf2/HO-2 axis. (a, b) Quantitative analysis of Nrf2 and HO-1 protein expression levels in the MC3T3-E1 cells exposed to different treatments. (c) Representative images showing Nrf2 immunofluorescence. (d) Quantitative analysis of Nrf2 fluorescence intensity in the cytoplasm and nucleus. Scale bar: 20 *μ*m. ^∗^*P* < 0.05 and ^∗∗^*P* < 0.01.

Oxidative stress and mitochondrial dysfunction inhibit osteogenic differentiation and mineralization. In this study, pretreatment with ADSCs-Exos restored ALP activities and mineralization compared to the Dex group (Figures 5(a) and 5(b)), and quantitative analysis of ALP- and ARS-positive area normalized to the control group using ImageJ software (Figures 5(c) and 5(d)). Meanwhile, western blot showed that ADSCs-Exos reversed the effects of Dex on osteogenic transcription factors Runx2, Bmp2, and Opn (Figures 5(e) and 5(f)). To determine whether ADSCs-Exos exert their effects on Dex-induced oxidative injury via Nrf2/HO-1, various indicators were assessed. Western blot showed that Dex exposure suppressed the activations of Nrf2 and HO-1, compared with the PBS group (Figures 6(a) and 6(b)). In contrast, ADSC-Exo pretreatment promoted Nrf2 and HO-1 activation. Fluorescence staining of MC3T3-E1 cells showed the red fluorescence of Nrf2 in both the nucleus and cytoplasm of the PBS group. However, in the Dex group, the red fluorescence of Nrf2 was significantly reduced and confined to the nucleus. Pretreatment with ADSCs-Exos significantly promoted the fluorescence intensity of Nrf2, especially in the nucleus (Figures 6(c) and 6(d)). These results show that ADSCs-Exos inhibit oxidative damage to MC3T3-E1 cells after Dex by promoting the nuclear translocation of Nrf2 and activating downstream HO-1.

### 3.6. The Nrf2/HO-1 Axis Mediated the Osteoprotective Effects of ADSCs-Exos

**Figure 7:**
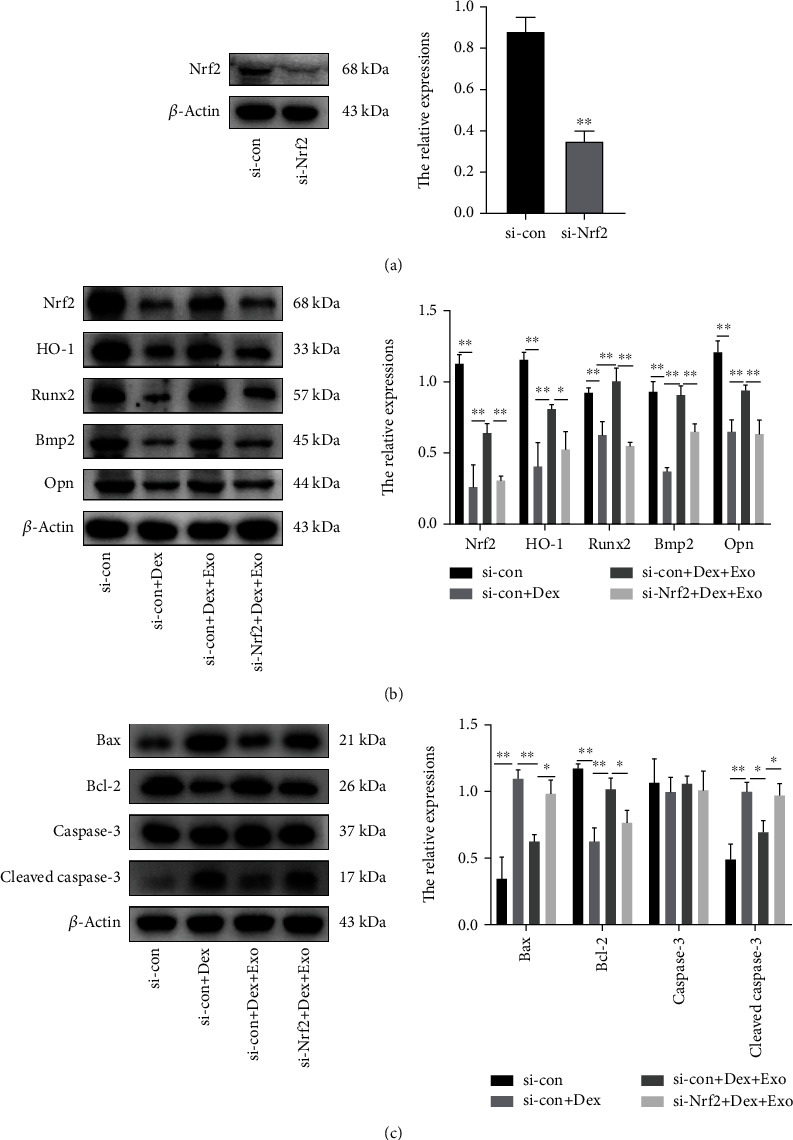
The Nrf2/HO-1 pathway mediated the regulatory effects of ADSCs-Exos on oxidative damage induced by Dex. (a) Expression of Nrf2 proteins in cells. (b) Immunoblot showing Nrf2, HO-1, Runx2, Bmp2, and Opn expression level in the cells exposed to different treatments. (c) Immunoblotting results showing the expression level of apoptosis-related proteins in MC3T3-E1 cells exposed to different treatments. ^∗^*P* < 0.05 and ^∗∗^*P* < 0.01.

To verify that the involvement of the Nrf2/HO-1 axis in ADSCs-Exos mediated osteoprotective effects against Dex-induced oxidative damage, Nrf2-siRNA knockdown of Nrf2 expression was performed.  Figure 7(a) shows that Nrf2 expressions were significantly suppressed after transfection. ADSCs-Exos blocked the osteogenic inhibition of MC3T3-E3 cells by Dex, while si-Nrf2 transfection partially reversed the effect of ADSCs-Exos (Figure 7(b)). In parallel, si-Nrf2 transfection downregulated the antiapoptotic effect of ADSCs-Exos (Figure 7(c)). Taken together, ADSCs-Exos protect the osteoblast function through Nrf2 and activation of its downstream pathways.

### 3.7. GIOP Animal Models

**Figure 8:**
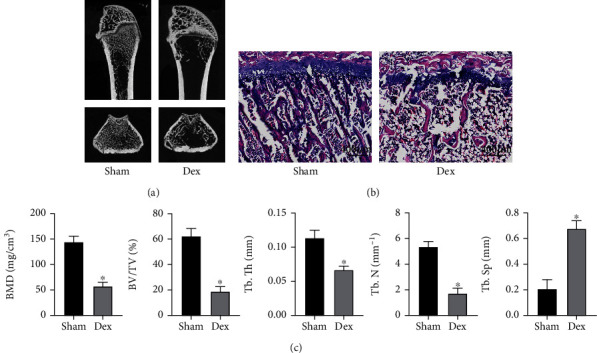
Establishment of the GIOP model. (a) Representative micro-CT images showing the distal femur in Sham and Dex-treated rats. (b) Representative HE staining of the distal femur in Sham and Dex-treated rats. Scale bar: 400 *μ*m. (c) The BMD, BV/TV, Tb.Th, Tb.N, and Tb.Sp values in the Sham and Dex groups. ^∗^*P* < 0.05.

After 4 weeks of intraperitoneal injection of the same volume of 2.5 mg/kg Dex or PBS, 5 rats were sacrificed in each group and distal femoral trabeculae structures analyzed by micro-CT and HE staining. The micro-CT shows that compared to Sham group, distal femoral trabeculae were significantly reduced in Dex group (Figures 8(a) and 8(c)). HE staining showed comparable results, confirming successful establishment of GIOP models (Figures 8(b)).

### 3.8. Therapeutic Effects of ADSCs-Exos in GIOP Rat Models

**Figure 9:**
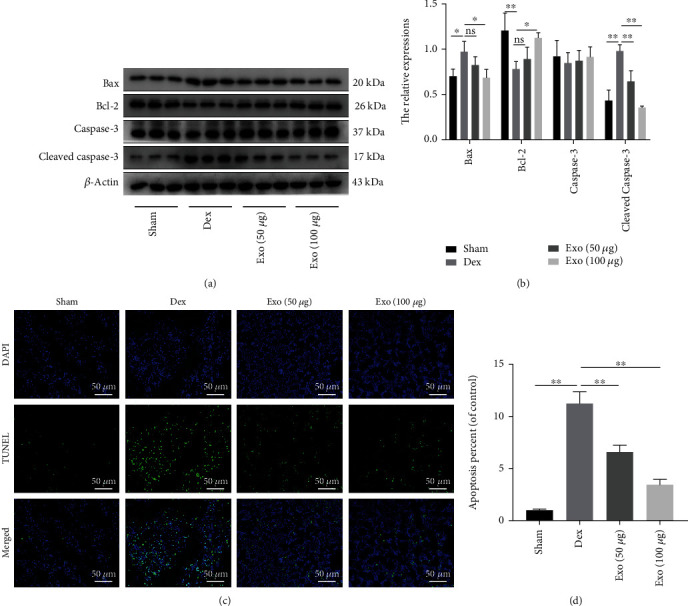
ADSCs-Exos inhibited oxidative stress and attenuated apoptosis in bone tissue after Dex. (a, b) Quantitative analysis of apoptosis-related proteins Bax, Bcl-2, and cleaved caspase-3. (c, d) Representative TUNEL staining and their quantitative analysis in the distal femur of rats. Apoptosis-positive cells were measured by TUNEL (green). Scale bar: 50 *μ*m. ^∗^*P* < 0.05 and ^∗∗^*P* < 0.01.

To assess the therapeutic effects of ADSCs-Exos in GIOP rat models, they were injected with ADSCs-Exos (50 or 100 *μ*g/200 *μ*l PBS three times a week for 5 weeks) or vehicle (PBS). First, the levels of apoptosis-related proteins were assessed to establish the effects of ADSCs-Exos on apoptosis in bone tissues after Dex. Dex significantly elevated proapoptotic protein (Bax and cleaved caspase-3) levels and downregulated antiapoptotic protein (Bcl-2) levels in bone tissues, compared with Sham group. However, treatment of rats with ADSCs-Exos reversed the changes in apoptosis-related protein levels (Figures 9(a) and 9(b)). In bone tissues, compared to Sham group, the number of TUNEL-positive cells was increased in the Dex group but was markedly decreased in ADSC-Exo-treated rats (Figures 9(c) and 9(d)). Thus, ADSCs-Exos inhibited Dex-induced oxidative injury-mediated apoptosis in bone tissues.

**Figure 10:**
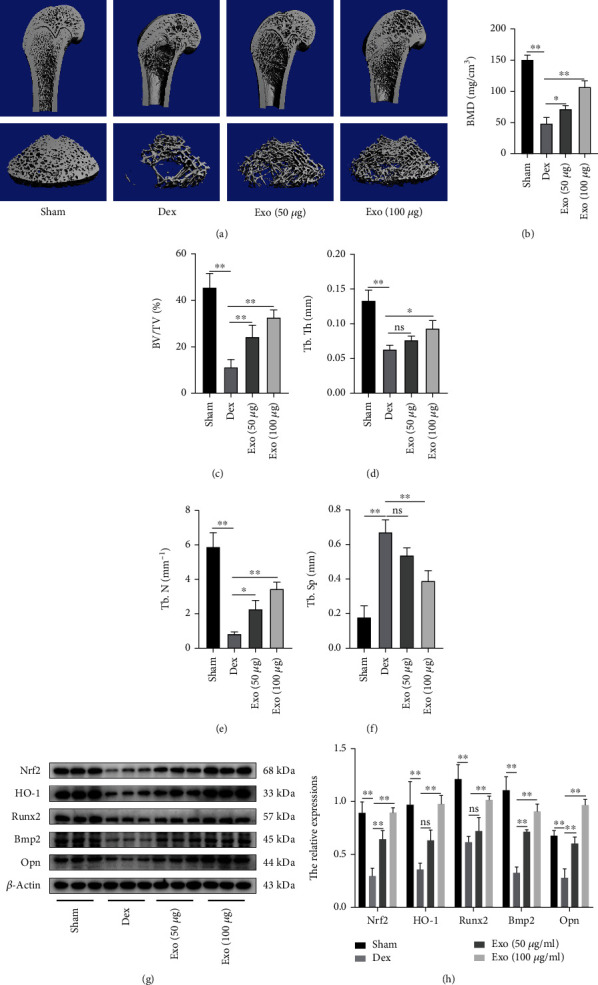
The therapeutic effect of ADSCs-Exos in the GIOP rat model. (a) Representative micro-CT reconstruction images of the distal femur of rats from the Sham, Dex, Exo (50 *μ*g), and Exo (100 *μ*g) groups. (b–f) Quantitative analysis of morphometric parameters of distal femoral. (g, h) Nrf2, HO-1, Runx2, Bmp2, and Opn expressions and their quantitative analysis. ^∗^*P* < 0.05 and ^∗∗^*P* < 0.01.

Micro-CT showed a significant recovery of bone trabeculae in the ADSC-Exo (50 or 100 *μ*g) group, compared with the Dex group (Figures 10(a)). To quantify these changes, trabecular bone microarchitectures in femora were analyzed and expressed as BMD, BV/TV, Tb.Th, Tb.N, and Tb.Sp. The higher dose of ADSC-Exo treatment significantly upregulated BMD, BV/TV, Tb.Th, as well as Tb.N, and downregulated Tb.Sp, compared to Dex and Exo (50 *μ*g) groups (Figures 10(b)–10(f)). Western blot revealed that Exo (50 and 100 *μ*g) elevated the levels of Nrf2 and HO-1 as well as those of osteogenic proteins (Runx2, Bmp2, and Opn) in the distal femur of rats, compared with the Dex group (Figures 10(g) and 10(h)).

**Figure 11:**
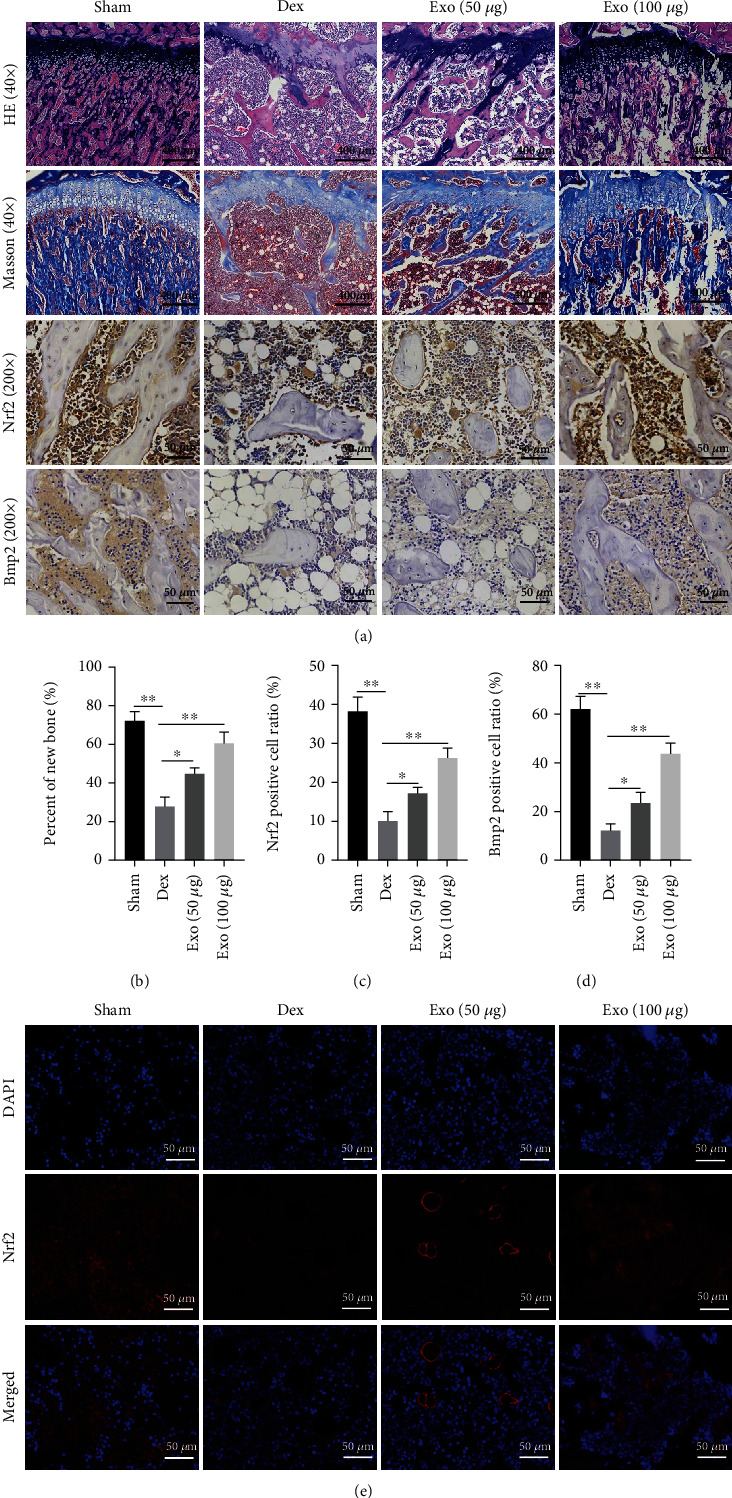
The therapeutic effect of ADSCs-Exos in GIOP rats. (a) Representative HE, Masson, and immunohistochemical staining images of Nrf2 and Bmp2. (b) Quantitative analysis of new bone formation rate in different groups. (c, d) IHC was quantified as the number of positive cells in stained tissue. (e) Representative images showing Nrf2 immunofluorescence. Scale bar: 50 *μ*m. ^∗^*P* < 0.05 and ^∗∗^*P* < 0.01.

Analysis of femoral epiphysis showed that findings from HE staining were comparable to those from micro-CT and that Exo treatment significantly reversed the Dex-induced destruction of the femoral trabeculae. The Masson staining and its quantitative analysis showed that Dex decreased the rate of new bone formation, compared to Sham group, while Exo restores the inhibitory effects of Dex on new bone formation in the distal femur of rats. Meanwhile, fluorohistochemical analysis showed that Nrf2 and Bmp2 protein expressions in bone tissues were lowest in the Dex group, followed by Exo (50 *μ*g) group, Exo (100 *μ*g) group, and Sham group (Figures 11(a)–11(d)). Immunofluorescence of Nrf2 in bone tissues showed comparable findings to those of immunohistochemistry. Thus, Exo is potentially therapeutic against GIOP (Figures 11(e)).

## 4. Discussion

In this study, we explored the role of ADSCs-Exos in oxidative damage after Dex and its potential mechanisms. First, we demonstrated the protective effects of ADSCs-Exos against oxidative stress, mitochondrial dysfunction, elevated ROS levels, and osteogenic injury after Dex in osteoblasts. ADSCs-Exos significantly activated the Nrf2/HO-1 axis after Dex exposure, while si-Nrf2 reversed the protective effects of ADSCs-Exos. Then, we established a classical animal model of glucocorticoid-induced osteoporosis, in which ADSC-Exo treatment attenuated Dex-induced apoptosis in bone tissues and inhibited bone loss. Thus, ADSCs-Exos can attenuate Dex-induced ROS accumulation and oxidative damage via regulating Nrf2/HO-1 levels, thereby inhibiting apoptosis and bone loss, which may be a new potential approach for treatment of GIOP.

Oxidative stress is strongly associated with development of osteoporosis and can result in extensive cell damage, including cell necrosis, autophagy, and apoptosis [26–28]. Moreover, ROS overproduction can disrupt mineral tissue homeostasis and bone remodeling by triggering oxidative stress [29, 30]. Glucocorticoids are the cornerstone for treatment of immune, oncologic, and allergic diseases. About 1-2% of the global population is on long-term glucocorticoid therapy [31]. Dex, a synthetic glucocorticoid, induces oxidative stress and ROS accumulation, leading to mitochondrial dysfunction, promoting apoptosis, and accelerating osteoporosis [32, 33]. In this study, treatment of MC3T3-E3 cells with Dex reduced cell viability, intracellular ROS accumulation, downregulation of mitochondrial membrane potential, and antiapoptotic protein (Bcl-2) levels, followed by increased apoptosis, as well as caspase activities, and decreased osteogenic capacity.

MSCs are a group of cells with the ability to self-renew and differentiate, which are involved in various physiological and pathological processes such as tissue regeneration, wound healing, and tumorigenesis [34]. In recent years, concerns have been raised about the safety of MSCs for clinical use. Study shows that MSCs after long-term culture increased the potential risk to develop tumors, because of dysregulation of cell cycle-related genes and chromosomal instability [35]. At the same time, risks such as cell dedifferentiation and immune rejection further limit the clinical application of MSCs [36]. Over the past decade, researchers have found that extracellular vesicles derived from MSCs have shown similar therapeutic effects to parent cells in some diseases [37]. Compared to MSCs, cell-free therapy of extracellular vesicles offers the advantages of immune silence, nononcogenicity, high stability, cell- and tissue-specific homing, and absence of vascular obstruction [38]. Extracellular vesicles are secreted by various tissues and cells and have membrane vesicle structures surrounded by a lipid bilayer [39]. These vesicles are classified into exosomes (30-150 nm, ultracentrifugation 100,000 × g pellet fraction), microvesicles (100-1000 nm, medium speed centrifugation 20,000 × g pellet), and apoptotic vesicles (500-5000 nm, low-speed centrifugation 2000 × g pellet) [40]. At present, it is a very challenging task to separate the extracellular vesicles according to their classification by centrifugal force. The exosomes have the smallest average particle size, the highest homogeneity, the most complex composition, and the most diverse functions. Therefore, exosomes have the highest application value and have been most intensively researched and widely applied. People mostly refer to extracellular vesicles as exosomes and often confuse exosomes with extracellular vesicles.

As an important bioactive substance secreted by ADSCs into the extracellular space, ADSCs-Exos exhibit regenerative functions in many diseases. Li et al. found that ADSCs-Exos accelerated the proliferation and angiogenesis of endothelial progenitor cells under high-glucose conditions, which facilitated skin wound healing [23]. Yang et al. reported that after oxygen-glucose deprivation (OGD), ADSCs-Exos promoted the migration and angiogenesis of brain microvascular endothelial cells (BMECs) *in vitro* [41]. ADSCs-Exos have been shown to exhibit anti-inflammatory, antiaging, and immunomodulatory functions, even in the absence of ADSCs. For example, ADSCs-Exos attenuated inflammation as well as apoptosis and promoted neovascularization after ischemia-reperfusion [42]. MiR-126-modified ADSCs-Exos downregulate hypoxia-induced inflammatory factor expressions and reduces damage to cardiomyocytes [43]. In this study, we successfully isolated ADSCs-Exos and identified them by NTA, western blot, and TEM. In the NTA experiments, we found that the particle size range of precipitate was normally distributed between 80 and 200 nm, with a peak of 115.1 nm. Therefore, we are favored to consider that the effect of exosomes was analyzed in the work. Then, we confirmed their effectiveness against oxidative damage after Dex. It was found that ADSCs-Exos inhibited the apoptosis of MC3T3-E1 cells and restored their osteogenic capacity by downregulating intracellular oxidative stress and ROS clustering, restoring antiapoptotic proteins (Bcl-2), and downregulating proapoptotic proteins (Bax and cleaved caspase-3). Studies should elucidate on the importance of ADSCs-Exos in osteoporosis.

Nrf2, a crucial transcription factor, coordinates the activation of multiple cytoprotective and antioxidant enzymes by encoding cytoprotective genes when intracellular ROS accumulate, exerting intracellular defense mechanisms to combat oxidative stress [44]. In the cytoplasm, Nrf2 binds Kelch-like ECH-associating protein 1 (Keap1) and is rapidly degraded [45]. When cells undergo oxidative stress, Nrf2 separates from Keap1 and translocates to the nucleus, activating antioxidant response element (ARE) genes, such as HO-1 and NQO1 [45, 46]. Activation of the Nrf2/HO-1 axis can upregulate the levels of various antioxidant enzymes and protect cardiomyocytes from oxygen radical damage [47]. Exosomes from M2-polarized macrophages inhibit ROS and MDA production by activating Nrf2/HO-1 and protecting oxygen/glucose deprivation/normalization-induced neuronal injury [48]. Therefore, specific targeting of the Nrf2/HO-1 axis maybe a new therapeutic strategy for treatment of human diseases, such as Alzheimer's disease, diabetes, and hepatotoxicity [44, 46, 49, 50]. We found that ADSCs-Exos protected the MC3T3-E1 cells from oxidative damage and promoted osteogenic differentiation as well as mineralization by promoting the Nrf2-mediated expressions of HO-1. Western blot showed that ADSCs-Exos elevated Nrf2 levels and upregulated HO-1 protein expressions, suggesting the activation of Nrf2/HO-1. Immunofluorescence of MC3T3-E1 cells showed that ADSCs-Exos promoted the nuclear translocation of Nrf2. Besides, Nrf2 knockdown attenuated the protective effects of ADSCs-Exos, thereby confirming that the Nrf2/HO-1 axis mediates the ADSCs-Exos effects.

Under certain pathological conditions, an imbalance between bone formation and bone resorption may occur, which leads to the development of osteoporosis [51]. Dex induces osteoporosis development by impairing the functions of osteoblasts. Therefore, Dex-induced osteoporosis models are widely used in mechanistic studies *in vivo* [52]. To assess and analyze more bone volume, we selected the distal femur. As the gold standard for bone mass change, BMD is often used clinically for diagnosis of osteoporosis [53]. Consistent with the previous reports, BMD was decreased in the distal femur of Dex-treated rats, indicating incomplete bone mineralization [54]. We found that ADSCs-Exos ameliorated Dex-induced BMD loss and bone volume reduction, with a significant restoration of bone trabecular number and spacing. In addition, oxidative stress modulates osteoblast differentiation and increases apoptosis to inhibit bone formation [55]. Therefore, we detected the relevant indicators in vivo. We found similar effects to other antioxidants [56, 57], ADSCs-Exos reversed the increase in the number of apoptosis-positive cells as well as the levels of apoptosis-related proteins induced by Dex and restored of oxidative stress. BMP2 is an osteogenic factor that can promote osteogenic differentiation by activating downstream transcription factors such as Runx2 [58]. Immunohistochemical results showed that BMP2 and Nrf2 expressions were downregulated in the Dex group, while ADSC-Exo treatment enhanced BMP2 and Nrf2 expressions. Consistent with immunofluorescence assays, ADSCs-Exos delayed GIOP progression and recovered the *in situ* Nrf2 levels in rat models. These findings imply that intravenous administration of ADSCs-Exos restores oxidative stress homeostasis and promotes bone trabeculae formation in rats.

## 5. Conclusions

ADSCs-Exos can attenuate Dex-induced ROS aggregation in osteoblasts, mitochondrial dysfunction, and osteogenic damage. Meanwhile, Nrf2 expression was significantly increased in Dex-stimulated osteoblasts after pretreatment with ADSCs-Exos. Inhibition assays revealed that the antiapoptotic effects of ADSCs-Exos are dependent on Nrf2. *In vivo*, ADSCs-Exos attenuated the development of GIOP. However, we did not investigate what components in ADSCs-Exos exert antioxidative stress and promote bone regeneration, which is the next research direction of our team.

## Data Availability

The data used to support the findings of this study are available from the corresponding author upon request.

## Abbreviations

GIOP: Glucocorticoid-induced osteoporosis
Dex: Dexamethasone
BMD: Bone mineral density
ROS: Reactive oxygen species
ADSCs: Adipose-derived stem cells
ADSCs-Exos: Exosomes from adipose-derived stem cells
Nrf2: Nuclear factor erythroid 2-related factor 2
SOD: Superoxide dismutase
TEM: Transmission electron microscopy
NTA: Nanoparticle tracking analysis
HO-1: Heme oxygenase-1
siRNA: Small interfering RNA
DCFH-DA: Dichlorodihydro-fluorescein diacetate
MMP: Mitochondrial membrane potential
MDA: Malondialdehyde
Opn: Osteopontin
ARS: Alizarin red S
Micro-CT: Microcomputed tomographic
BV/TV: Bone volume per total volume
Tb.Th: Bone trabecular thickness
Tb.N: Trabecular number
Tb.Sp: Trabecular spacing
TUNEL: Terminal deoxynucleotidyl transferase-mediated end-of-cut labeling.
